# Safety, Tolerability, and Immunogenicity of the Pneumococcal Vaccines PPSV23 or PCV15 Co-Administered with a Booster Dose of mRNA-1273 SARS-CoV-2 Vaccine in Healthy Adults ≥50 Years of Age

**DOI:** 10.3390/vaccines13020192

**Published:** 2025-02-15

**Authors:** Tosin Omole, Enrique Pelayo, Aaron S. Weinberg, Spyros Chalkias, Zelalem Endale, Gretchen Tamms, Tina M. Sterling, Lori Good, Tulin Shekar, Morgan Johnson, Natalie Banniettis, Ulrike K. Buchwald, Alejandra Esteves-Jaramillo

**Affiliations:** 1 Merck & Co., Inc., Rahway, NJ 07065, USA; gretchen_tamms@merck.com (G.T.); tina_sterling@merck.com (T.M.S.); lori_good@merck.com (L.G.); tulin.shekar@merck.com (T.S.); morgan.johnson@merck.com (M.J.); natalie.banniettis@merck.com (N.B.); ulrike.buchwald@merck.com (U.K.B.); alex.esteves@merck.com (A.E.-J.); 2 Advanced Medical Research Institute, Miami, FL 33174, USA; epelayomd@amriresearch.com; 3 Carbon Health, North Hollywood, CA 91606, USA; aaron@carbonhealth.com; 4 Moderna, Cambridge, MA 02142, USA; spyros.chalkias@modernatx.com (S.C.); zelalem.endale@modernatx.com (Z.E.)

**Keywords:** COVID-19, immunogenicity, pneumococcal, safety, tolerability, vaccination, vaccine

## Abstract

Background/Objectives: *Streptococcus pneumoniae* with, or following, severe acute respiratory syndrome coronavirus 2 (SARS-CoV-2) infection has been associated with increased mortality, particularly in older adults. However, vaccination can be an effective preventative measure. This Phase 3 study (NCT05158140) assessed the immunogenicity and safety of co-administering the SARS-CoV-2 vaccine mRNA-1273 with the 23-valent pneumococcal polysaccharide vaccine (PPSV23) or the 15-valent pneumococcal conjugate vaccine (PCV15). Methods: Participants were healthy adults ≥50 years of age who had previously received a two-dose primary series of mRNA-1273 ≥5 months before the first study visit and may have received a booster dose of mRNA-1273 ≥4 months prior to the first study visit. Participants were randomized (1:1:1:1) to receive mRNA-1273 concomitantly with PPSV23 or PCV15 on Day 1 followed by placebo on Day 30, or sequentially with mRNA-1273 and placebo on Day 1 and PPSV23 or PCV15 on Day 30. The primary study endpoints were pneumococcal-serotype-specific opsonophagocytic activity (OPA) geometric mean titers (GMTs) and SARS-CoV-2-specific binding antibody GMTs at 30 days after vaccination, as well as safety and tolerability following vaccination. Results: In total, 850 adults participated in the study. Serotype-specific OPA GMTs at 30 days post-vaccination with PPSV23 or PCV15 were generally comparable between the concomitant and sequential groups. SARS-CoV-2-specific GMTs increased in all groups from pre-vaccination to 30 days post-vaccination with mRNA-1273, with a consistent response between concomitant and sequential groups. Safety profiles were comparable across study groups. Conclusions: Co-administration of mRNA-1273 with PPSV23 or PCV15 in healthy adults ≥50 years of age was immunogenic and well tolerated.

## 1. Introduction

The burden of disease caused by *Streptococcus pneumoniae* is high among adults ≥50 years of age and is highest in older adults ≥65 years of age [1,2,3]. Infection with severe acute respiratory syndrome coronavirus 2 (SARS-CoV-2), which results in coronavirus disease 2019 (COVID-19), can predispose individuals to bacterial infections and diseases, such as pneumococcal disease (PD) [4]. Pneumococcal infection can occur at the same time as an infection with SARS-CoV-2 (co-infection) or can occur afterwards as a secondary infection [4,5]; both have been associated with increased mortality compared with invasive PD (IPD) or COVID-19 alone [6]. This increased mortality can often be observed among at-risk populations, such as older adults or those with certain underlying chronic medical conditions [7]. In addition, studies have shown that individuals who experience milder symptoms of SARS-CoV-2 infection, such as fever, mild or moderate coughing, and shortness of breath, can have higher carriage rates of *S. pneumoniae* than those not infected with SARS-CoV-2 [8]. Furthermore, infection with SARS-CoV-2 can compromise the immune system and is considered a primary risk factor for IPD [8,9].

Pneumococcal polysaccharide and conjugate vaccines with varying serotype coverage are recommended by the US Centers for Disease Control and Prevention (CDC) for children ≤5 years of age, adults ≥50 years of age, and children and adults at increased risk of PD [10,11]. Pneumococcal vaccines have a long history of use and have been demonstrated to be immunogenic and effective, with a favorable safety profile [12]. These vaccines are the mainstay of disease prevention and have led to a substantial decrease in cases of IPD, respiratory disease burden, and healthcare utilization [13,14,15]. A widely used pneumococcal vaccine is the 23-valent pneumococcal polysaccharide vaccine (PPSV23; Pneumovax^®^ 23, Merck Sharp & Dohme LLC, a subsidiary of Merck & Co., Inc., Rahway, NJ, USA [MSD]). PPSV23 is indicated for the prevention of PD in older adults ≥50 years of age and individuals ≥2 years of age with certain medical conditions that can lead to an increased risk of PD. PPSV23 contains 23 different serotypes of *S. pneumoniae* (serotypes 1, 2, 3, 4, 5, 6B, 7F, 8, 9N, 9V, 10A, 11A, 12F, 14, 15B, 17F, 18C, 19A, 19F, 20, 22F, 23F, and 33F) [13,16,17,18,19]. A 15-valent pneumococcal conjugate vaccine (PCV15; VAXNEUVANCE™, MSD) is also available in many countries and is indicated in individuals ≥6 weeks of age for the prevention of IPD and pneumonia, and in individuals 6 weeks to ≤18 years of age for acute otitis media, caused by *S. pneumoniae* serotypes contained in the vaccine (1, 3, 4, 5, 6A, 6B, 7F, 9V, 14, 18C, 19A, 19F, 22F, 23F, and 33F) [15]. The CDC recommends that, in adults, administration of PCV15 should be followed by a single dose of PPSV23 1 year later [10,20].

After the novel SARS-CoV-2 virus was identified in 2019, an urgent need arose for vaccines to curb the rapidly spreading pandemic, reduce severe illness and mortality, and address the global health crisis [21]. The mRNA-1273 COVID-19 vaccine (SPIKEVAX^®^, Moderna Inc., Cambridge, MA, USA) received emergency use authorization (EUA) from the US Food and Drug Administration on 18 December 2020, for the immunization of adults ≥18 years of age. Almost a year later, the EUA was amended to include the use of an mRNA-1273 booster dose, administered ≥6 months after completion of the primary series [22]. mRNA-1273 is a nucleoside-modified messenger RNA (mRNA) vaccine containing single mRNAs encoding the prefusion-stabilized spike glycoprotein of ancestral SARS-CoV-2 (Wuhan-Hu-1). During the study period, the monovalent mRNA-1273 was indicated for active immunization to prevent COVID-19 caused by SARS-CoV-2 in individuals ≥12 years of age (SPIKEVAX^®^ [COVID-19 Vaccine, mRNA, 2024–2025 Formula], Moderna TX, Cambridge, MA, USA) [23].

The US Advisory Committee on Immunization Practices (ACIP) recommends that SARS-CoV-2 vaccines be co-administered with other vaccines [24]. The feasibility of co-administration of vaccines in older adults has been previously demonstrated [25,26]. Co-administration of vaccines helps increase vaccination coverage by reducing the number of vaccination visits, lowering costs, minimizing missed opportunities, improving compliance, and ensuring timely vaccine administration [27,28,29]. In a meta-analysis of 17 studies, co-administration of influenza and pneumococcal vaccines resulted in an additional 24% reduction in pneumonia and an additional 28% reduction in deaths in older adults compared with pneumococcal vaccination alone [30]. Furthermore, in a study of US adults who had received 13-valent pneumococcal conjugate vaccine (Prevnar13^®^, Pfizer Inc., New York, NY, USA) with or without PPSV23, based on the intervals recommended in the 2015 ACIP guidelines for those ≥65 years of age, pneumococcal vaccination was associated with a reduced risk of COVID-19 diagnosis, hospitalization, or fatal hospitalization [31].

This study was conducted to assess the safety, tolerability, and immunogenicity of mRNA-1273 administered concomitantly with a single dose of a pneumococcal vaccine (PPSV23 or PCV15), compared with sequential administration of the same vaccines, in healthy adults ≥50 years of age.

## 2. Methods

**Figure 1:**
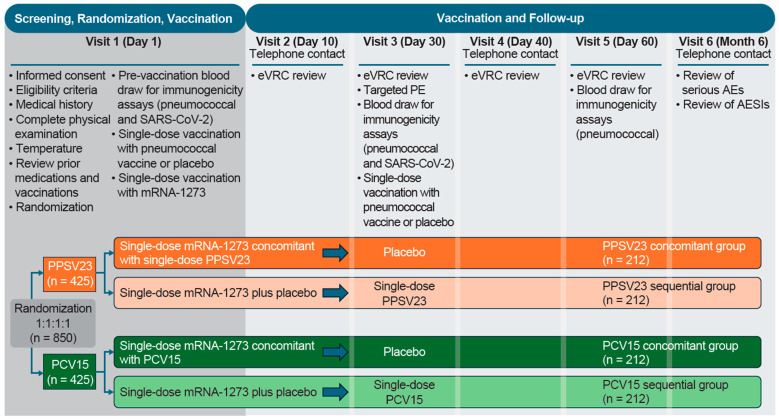
Study design. AE: adverse event; AESI: adverse event of special interest; eVRC: electronic vaccination report card; mRNA-1273: monovalent mRNA COVID-19 vaccine; PCV15: 15-valent pneumococcal conjugate vaccine; PE: physical examination; PPSV23: 23-valent pneumococcal polysaccharide vaccine; SARS-CoV-2: severe acute respiratory syndrome coronavirus 2.

This Phase 3, randomized, placebo-controlled, parallel-group, multicenter, double-blind study was conducted between 12 January 2022 and 27 October 2023, during the COVID-19 pandemic (Protocol V110-911; NCT05158140; Figure 1). Participants were enrolled at 44 sites across the United States and Puerto Rico (Table S1).

### 2.1. Participants, Randomization, and Blinding

Eligible participants were adults ≥50 years of age who had previously completed a two-dose primary series of mRNA-1273 ≥5 months before the receipt of the study vaccine at the first study visit. Participants with historic SARS-CoV-2 infection and those who had received a prior booster dose of mRNA-1273 were also included. The study was conducted amidst an evolving SARS-CoV-2 vaccine landscape and the protocol was amended to reflect changes in vaccine recommendations. Participants were excluded if they had a current SARS-CoV-2 infection, a known history of SARS-CoV-2 infection <3 months before receipt of the study vaccine at the first study visit, or if they had a history of myocarditis and/or pericarditis. Participants with underlying chronic conditions were assessed by the investigator to be in a stable condition. Detailed inclusion and exclusion criteria are provided in Table S2.

Participants were randomly assigned in a 1:1:1:1 ratio via a centralized interactive response technology system. Participants who received mRNA-1273 (50 µg/0.25 mL) concomitantly with either PPSV23 (refer to product label for dosage) or PCV15 (refer to product label for dosage) at the first study visit, followed by placebo at Visit 3, were in the PPSV23 concomitant group or PCV15 concomitant group, respectively. Participants who received mRNA-1273 with placebo at the first study visit, followed by either PPSV23 or PCV15 at Visit 3, were in the PPSV23 sequential group or PCV15 sequential group, respectively (Figure 1). Stratification factors included age (50–64 years, 65–74 years, and ≥75 years of age), history of pneumococcal vaccination (yes or no), receipt of prior mRNA-1273 booster dose following the two-dose primary series (yes or no), and history of prior SARS-CoV-2 infection (yes or no).

PPSV23, PCV15, and placebo were prepared and administered by an unblinded pharmacist or qualified personnel. The participant, investigator, and other personnel involved in study conduct or clinical evaluation were blinded. mRNA-1273 was provided open-label and was also prepared and administered by unblinded personnel.

This study was conducted in accordance with principles of Good Clinical Practice guidelines and was approved by the appropriate institutional review boards and regulatory agencies [32]. The institutional review board approved the protocol, protocol amendments, and informed consent form. All participants provided written informed consent before enrollment. The study protocol and statistical analysis plan are available online [33].

### 2.2. Immunogenicity Assessments

There were two primary immunogenicity endpoints in this study. The first was to evaluate the pneumococcal-serotype-specific opsonophagocytic activity (OPA) geometric mean titers (GMTs) at 30 days post-vaccination with PPSV23 or PCV15 in each of the four groups. The other primary endpoint was to evaluate SARS-CoV-2-specific binding antibody GMTs at 30 days post-vaccination with mRNA-1273 in each of the four groups. Data from both sequential groups were combined for the SARS-CoV-2 immunogenicity analysis.

Secondary endpoints included assessing the serotype-specific geometric mean fold rises (GMFRs) and the proportions of participants with a ≥4-fold rise from baseline (pre-vaccination) to 30 days post-vaccination for OPA responses in each group. For SARS-CoV-2-specific binding antibody responses, the GMFRs and proportions of participants with a ≥4-fold rise from baseline to 30 days post-vaccination with mRNA-1273 were assessed in each group. The immunogenicity analyses were based on the per-protocol population, which comprised all randomized participants without deviations from the protocol that could have substantially affected immunogenicity measurements.

Immune responses were measured for the PCV15 serotypes (1, 3, 4, 5, 6A, 6B, 7F, 9V, 14, 18C, 19A, 19F, 22F, 23F, and 33F) in a validated multiplex opsonophagocytic assay. This assay measured 14 of the 23 pneumococcal serotypes in PPSV23 (1, 3, 4, 5, 6B, 7F, 9V, 14, 18C, 19A, 19F, 22F, 23F, and 33F). A validated ligand-binding assay specific to the SARS-CoV-2 spike protein was used to measure vaccine-induced responses to mRNA-1273.

### 2.3. Safety Assessments

The primary safety objective was the assessment of safety and tolerability of a booster dose of mRNA-1273 administered concomitantly or sequentially with a single dose of pneumococcal vaccine (PPSV23 or PCV15). Primary safety endpoints included solicited injection-site adverse events (AEs), solicited systemic AEs, and vaccine-related serious AEs (SAEs). Participants were followed for 7 days post-vaccination for the number of solicited injection-site and systemic AEs. All solicited injection-site AEs (from Day 1 through Day 7 post-vaccination) were considered to be vaccine-related. All SAEs and AEs of special interest (AESIs), discontinuations of study intervention due to an AE, and deaths were collected from Day 1 through to study completion. AESIs were consistent with the regulatory guidance for the safety evaluation of mRNA-1273 and are listed in Table S3. Post-vaccination body temperature measurement was the only vital sign collected, and the temperatures were recorded after each vaccination on Days 1–7 using an electronic vaccination report card. All AEs and SAEs were assessed for overall intensity grade by the investigator and were recorded along with AE duration in days. Instances of injection-site erythema and swelling occurring on Days 1–7 were categorized as mild (2.5–5.0 cm), moderate (5.1–10.0 cm), or severe (>10 cm). Safety analyses were based on the all-participants-as-treated population, which included all randomized participants who received at least one dose of the study vaccine.

### 2.4. Statistical Analyses

On 31 August 2022, EUA for monovalent COVID-19 vaccines was withdrawn in favor of bivalent COVID-19 vaccine boosters. This change resulted in prematurely concluding enrollment [34,35]. Therefore, the planned enrollment of 1300 participants was changed to approximately 850 participants. Owing to this change, the study was descriptive in nature and not based on formal statistical hypothesis testing.

Evaluation of the OPA GMTs and the binding antibody GMTs at 30 days post-vaccination in each group included descriptive summaries and within-group 95% confidence intervals (CIs) for each vaccination group. The point estimates were calculated by exponentiating the estimates of the mean of the natural log values, and the within-group CIs were derived by exponentiating the CIs of the mean of the natural log values based on the t-distribution.

Reverse cumulative distribution curves were used to show the distribution of serotype-specific OPA titers and SARS-CoV-2-specific binding antibody titers for PPSV23 and PCV15 concomitant and sequential groups.

The safety analysis followed a tiered approach. For the Tier 2 safety endpoints, point estimates, with corresponding within-group 95% CIs based on the exact binomial method proposed by Clopper and Pearson [36], were provided for the proportions of participants with events following any vaccination. Clinical laboratory evaluations were not prespecified for this study. The Medical Dictionary for Regulatory Activities (MedDRA) version 26.0 was used for this study.

## 3. Results

### 3.1. Study Population

**Figure 2:**
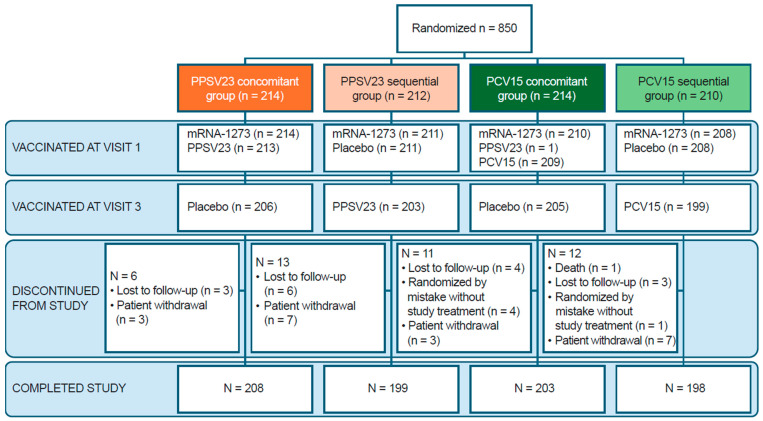
CONSORT flow diagram. Each participant was counted once. Two participants received study interventions other than what they were randomized to receive. In the PPSV23 concomitant group, one participant received two doses of mRNA-1273 at the first study visit. In the PCV15 concomitant group, one participant received PPSV23 and mRNA-1273 at the first study visit. Enrollment was closed on 31 August 2022, following EUA of the bivalent COVID-19 vaccine boosters and revocation of the EUA for monovalent COVID-19 vaccine boosters. At the time enrollment was closed, 850 participants were enrolled. As of the final database lock, 850 participants were randomized. COVID-19: coronavirus disease 2019; EUA: emergency use authorization; mRNA-1273: monovalent mRNA COVID-19 vaccine; PCV15: 15-valent pneumococcal conjugate vaccine; PPSV23: 23-valent pneumococcal polysaccharide vaccine.

A total of 850 participants were randomized: 212 participants in the PPSV23 sequential group, 210 participants in the PCV15 sequential group, and 214 participants in each of the PPSV23 and PCV15 concomitant groups (Figure 2). Most randomized participants received at least one dose of study vaccine and completed the study (≥93.9%). The most common reasons for discontinuing the study across all groups were participant withdrawal (2.4%) and loss to follow-up (1.9%). Most randomized participants were included in the immunogenicity analyses; in the PPSV23 sequential and PCV15 sequential groups, ≥87.3% and ≥87.6% were included at one or more timepoints, respectively. In addition, ≥85.8% and ≥87.6% were included for both baseline and post-vaccination timepoints, respectively.

Baseline demographic characteristics were comparable across all groups (Table 1 and Table S4). The mean age of participants was 60.4 years, with 73.4% of participants being in the 50–64-years-old age category; 56.9% were female, 77.9% were white, and 43.2% were of Hispanic or Latino ethnicity. Participants across all four groups generally had comparable pre-existing medical conditions (Table S5). No participants had a prior history of infection with SARS-CoV-2.

### 3.2. Immunogenicity

Serotype-specific OPA GMTs at 30 days post-vaccination were generally comparable between the PPSV23 concomitant and sequential groups and PCV15 concomitant and sequential groups (Table 2). A trend was observed in which the serotype-specific OPA GMTs for some serotypes were numerically lower in the PPSV23 and PCV15 concomitant groups compared with the PPSV23 or PCV15 sequential groups, respectively. The distribution of serotype-specific OPA titers was generally comparable between all groups, as demonstrated by the reverse cumulative distribution curves (Figures S1 and S2).

The observed serotype-specific GMFRs and the proportions of participants with a ≥4-fold rise in GMFRs from baseline to 30 days post-vaccination with PPSV23 and PCV15 were generally comparable for most serotypes in the PPSV23 and PCV15 concomitant groups compared with the PPSV23 and PCV15 sequential groups (Tables S6 and S7).

SARS-CoV-2-specific binding antibody GMTs at 30 days post-vaccination with mRNA-1273 were numerically lower in the PPSV23 and PCV15 concomitant groups compared with the PPSV23 and PCV15 sequential groups (Table 3). The distribution of SARS-CoV-2-specific binding antibody titers was similar between the PPSV23 sequential and concomitant groups, as well as the PCV15 sequential and concomitant groups, as shown by reverse cumulative distribution curves (Figure S3).

The observed SARS-CoV-2 binding antibody titer GMFRs and the proportions of participants with a ≥4-fold rise in GMFRs from baseline to 30 days post-vaccination with mRNA-1273 were generally comparable in the PPSV23 and PCV15 concomitant groups compared with the PPSV23 and PCV15 sequential groups (Tables S6 and S7).

### 3.3. Safety

**Figure 3:**
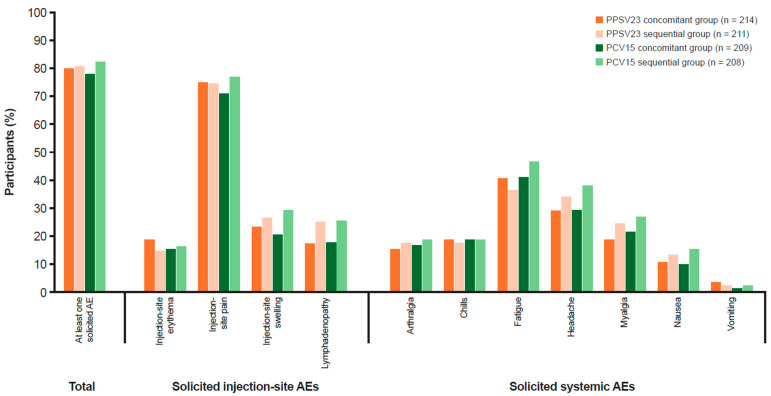
Participants with solicited AEs (incidence >0% in one or more group) in the all-participants-as-treated population following any vaccination. Every participant was counted a single time for each AE. Injection-site erythema, injection-site pain, injection-site swelling, lymphadenopathy, arthralgia, fatigue, headache, myalgia, nausea, vomiting, and chills were solicited from Day 1 to Day 7 following vaccination. MedDRA version 26.0 was used. AE: adverse event; MedDRA: Medical Dictionary for Regulatory Activities; PCV15: 15-valent pneumococcal conjugate vaccine; PPSV23: 23-valent pneumococcal polysaccharide vaccine.

The majority of participants in the PPSV23 groups (81.4%) and PCV15 groups (81.1%) reported at least one AE following any vaccination (Table 4). The proportions of participants with AEs, including injection-site AEs, systemic AEs, vaccine-related AEs, and SAEs, were generally comparable across all groups. Post-vaccination, the most common AEs in the PPSV23 and PCV15 groups were solicited AEs (≥79% and ≥78%, respectively; Figure 3), which were similar in frequency in all groups, and most events were classified as mild (Grade 1) or moderate (Grade 2) in intensity. The incidence of severe solicited AEs (Grade 3) was low in the PPSV23 and PCV15 groups (≤4.3% and ≤5.8%, respectively). Injection-site pain was the most frequently reported solicited AE in the PPSV23 and PCV15 groups (74.6% and 73.9%, respectively; Table S8).

The proportions of participants with systemic AEs were generally comparable between the PPSV23 concomitant (57.9%) and sequential (54.2%) groups, and between the PCV15 concomitant (53.1%) and sequential (54.2%) groups. The proportions of participants with vaccine-related systemic AEs were similar between the PPSV23 concomitant (55.6%) and sequential (51.2%) groups, and between the PCV15 concomitant (53.1%) and sequential (59.6%) groups, following any vaccination. The incidence of SAEs was low in both the PPSV23 (<1%) and PCV15 (<3%) groups, and none were considered by the investigator to be related to study vaccine.

One participant (0.2%) discontinued from the PPSV23 concomitant group due to a non-serious AE of injection-site induration, which was considered by the investigator to be vaccine-related. Two (0.5%) participants (both from the PCV15 sequential group) discontinued due to SAEs, which were not considered by the investigator to be vaccine-related; one participant had an SAE of pancreatic carcinoma and the other had an SAE, which was also classified as an AESI, of cardiac arrest on Day 8, resulting in death. There were no AESIs or deaths due to AEs in the PPSV23 groups. The frequency of solicited maximum body temperature measurements was similar between the PPSV23 groups and the PCV15 groups, with <100.4 °F (38.0 °C) being reported in >94% of participants in each group.

## 4. Discussion

The results from this study support the co-administration of mRNA-1273 with either PPSV23 or PCV15. Serotype-specific OPA GMTs at 30 days post-vaccination were generally comparable when PPSV23 or PCV15 were administered concomitantly or sequentially with mRNA-1273. Concomitant administration of mRNA-1273 with either PPSV23 or PCV15 could alter antigen uptake and the function of antigen-presenting cells, potentially influencing the immunogenicity of one or both vaccines compared with administering mRNA-1273 alone [37,38]. The SARS-CoV-2 antibody responses were lower when mRNA-1273 was administered concomitantly with PPSV23 or PCV15, compared with mRNA-1273 + placebo. However, this difference is unlikely to be clinically meaningful, given the robust GMTs observed overall. Furthermore, reverse cumulative distribution curves for SARS-CoV-2 binding antibody titers, binding antibody GMFRs, and the proportions of participants with ≥4-fold rises in binding antibody GMTs from baseline to 30 days post-vaccination with mRNA-1273 were generally comparable between the groups.

The safety results from this study showed that co-administration of PPSV23 or PCV15 with mRNA-1273 was well tolerated, and the safety profiles of concomitant administration were consistent with those of the individual vaccines, as observed in other studies [39,40,41,42,43]. The overall proportions of participants with AEs were generally comparable between the groups.

Further evidence to support the feasibility and potential benefits of co-administration of a pneumococcal vaccine with a COVID-19 vaccine was demonstrated in a Phase 3 study [44]. The safety and immunogenicity profiles of concomitant administration of 20-valent pneumococcal conjugate vaccine (Prevnar20^®^, Pfizer Inc., New York, NY, USA) and BNT162b2 (Comirnaty^®^; Pfizer-BioNTech) were comparable to those observed when each vaccine was administered alone [44]. The co-administration of pneumococcal and COVID-19 vaccines could be a strategy to increase vaccination rates by maximizing opportunities to vaccinate populations at-risk and by helping to increase the likelihood that individuals are fully vaccinated at the appropriate age, ultimately having a positive impact on public health [26,29,30]. Several studies have assessed the co-administration of COVID-19 vaccines and influenza vaccines [25,29,45,46]. The studies reported no major safety issues associated with co-administration, and AEs were typically of mild or moderate severity and were self-limiting. Co-administration did not significantly affect the immunogenicity of either the influenza or COVID-19 vaccines [25,29,45,46]. A systematic literature review reported that, in studies that assessed the acceptability of co-administration, the majority of individuals expressed willingness to receive both vaccines [29]. This growing body of evidence supports the benefits of administering COVID-19 vaccines alongside other vaccines. Therefore, there is a strong rationale for raising awareness about the safety of vaccine co-administration, which could enhance compliance with vaccine recommendations [27]. Furthermore, concomitant administration of vaccines ensures that individuals facing challenges in accessing care receive full protection [27,29]. Co-administration decreases the number of consultations and avoids scheduling multiple vaccination appointments that can often be missed and lead to higher rates of attrition [28,29].

The limitations of the study included that it was descriptive in nature and not based on formal statistical hypothesis testing, as enrollment closed early [34,35]. Although formal statistical comparisons were not made, serotype-specific OPA GMTs and SARS-CoV-2-specific GMTs for concomitant and sequential groups demonstrated generally comparable immunogenicity, with overlapping 95% CIs for most serotypes tested, suggesting limited changes in immunogenicity when administered concomitantly. While it is not expected that any numerical differences observed between groups are clinically meaningful, real-world evidence is required to confirm. As this study evaluated the original mRNA-1273 formulation, it is important to note the findings may not fully apply to the updated mRNA-1273 formulations or other pneumococcal vaccines not tested. However, clinical trial and safety surveillance data support the concomitant administration of pneumococcal vaccines with these updated formulations, showing comparable safety and immunologic profiles to the original mRNA-1273 vaccine [47,48,49]. Another limitation was that the study assessed individuals who previously received the mRNA-1273 vaccine, while, in practice, many individuals receive a mix of SARS-CoV-2 vaccines from different manufacturers. These factors restrict the generalizability of the findings to broader vaccine strategies [30,50].

## 5. Conclusions

This study provides evidence that the co-administration of mRNA-1273 with PPSV23 or PCV15 in healthy adults ≥50 years of age is immunogenic, with a safety profile comparable to administering mRNA-1273 with a placebo. These results add to the growing evidence that COVID-19 vaccines have a good safety profile and are effective when administered concomitantly with other vaccines [44]. These findings may inform future vaccination recommendations or vaccination guidelines, enhance vaccine program implementation, and potentially help to increase coverage rates for other licensed vaccines.

## Figures and Tables

**Table 1:** Baseline characteristics and demographics.

Participants in Population	PPSV23 Concomitant Group (n = 214)	PPSV23 Sequential Group (n = 211)	PCV15 Concomitant Group (n = 210)	PCV15 Sequential Group (n = 208)
**Sex, n (%)**				
Male	107 (50.0)	80 (37.9)	98 (46.7)	78 (37.5)
Female	107 (50.0)	131 (62.1)	112 (53.3)	130 (62.5)
**Mean age, years (range)**	60.5 (50–93)	60.5 (50–89)	60.5 (50–88)	60.4 (50–86)
**Age category**				
50–64 years	158 (73.8)	155 (73.5)	153 (72.9)	153 (73.6)
65–74 years	41 (19.2)	42 (19.9)	43 (20.5)	43 (20.7)
≥75 years	15 (7.0)	14 (6.6)	14 (6.7)	12 (5.8)
**Race, n (%)**				
White	164 (76.6)	173 (82.0)	163 (77.6)	157 (75.5)
Black or African American	41 (19.2)	32 (15.2)	36 (17.1)	40 (19.2)
Asian	4 (1.9)	3 (1.4)	7 (3.3)	4 (1.9)
Multiple	5 (2.3)	3 (1.4)	3 (1.4)	5 (2.4)
American Indian or Alaska Native	0	0	1 (0.5)	2 (1.0)
**Ethnicity, n (%)**				
Not Hispanic or Latino	127 (59.3)	111 (52.6)	122 (58.1)	116 (55.8)
Hispanic or Latino	87 (40.7)	98 (46.4)	88 (41.9)	91 (43.8)
Not reported	0	2 (0.9)	0	1 (0.5)
**Prior medications, n (%)**				
At least one prior medication	154 (72.0)	162 (76.8)	160 (76.2)	148 (71.2)
No prior medication	60 (28.0)	49 (23.2)	50 (23.8)	60 (28.8)
**Prior vaccinations, n (%)**				
At least one prior vaccination	0	1 (0.5) ^†^	1 (0.5) ^‡^	0
No prior vaccinations	214 (100.0)	210 (99.5)	209 (99.5)	208 (100.0)
**Concomitant medications, n (%)**				
At least one concomitant medication	164 (76.6)	173 (82.0)	167 (79.5)	162 (77.9)
No concomitant medications	50 (23.4)	38 (18.0)	43 (20.5)	46 (22.1)
**Concomitant vaccinations, n (%)**				
At least one concomitant vaccination	4 (1.9)	1 (0.5)	7 (3.3)	4 (1.9)
No concomitant vaccinations	210 (98.1)	210 (99.5)	203 (96.7)	204 (98.1)

Each participant is counted once per parameter. ^†^ A participant in the PPSV23 sequential group had previously received a vaccine for tetanus. ^‡^ A participant in the PCV15 group had previously received an inactivated vaccine for influenza. PCV15: 15-valent pneumococcal conjugate vaccine; PPSV23: 23-valent pneumococcal polysaccharide vaccine.

**Table 2:** Primary immunogenicity endpoint: serotype-specific OPA responses at 30 days post-vaccination.

	PPSV23 Concomitant Group (n = 214)		PPSV23 Sequential Group (n = 211)		PCV15 Concomitant Group (n = 210)		PCV15 Sequential Group (n = 208)	
Pneumococcal Serotype	n	Observed Response (95% CI) ^†^	n	Observed Response (95% CI) ^†^	n	Observed Response (95% CI) ^†^	n	Observed Response (95% CI) ^†^
1	182	236.0 (177.0–314.6)	177	253.5 (188.4–341.1)	186	208.3 (160.8–269.9)	174	265.6 (203.4–346.7)
3	167	174.3 (138.6–219.2)	164	284.0 (224.1–359.9)	174	237.5 (197.5–285.6)	155	338.4 (275.3–416.0)
4	182	1384.5 (1110.7–1725.9)	165	1704.4 (1370.6–2119.5)	181	1586.0 (1296.5–1940.2)	176	1758.6 (1419.4–2178.8)
5	189	423.4 (324.9–551.7)	181	376.3 (282.6–501.2)	190	447.0 (341.7–584.8)	180	505.5 (382.7–667.8)
6A	–	–	–	–	176	4345.3 (3490.0–5410.1)	162	5787.1 (4468.4–7495.0)
6B	182	1336.7 (1038.2–1721.1)	171	1326.8 (1016.7–1731.4)	173	3655.5 (2939.1–4546.6)	158	4992.9 (3896.2–6398.2)
7F	184	2614.4 (2123.8–3218.4)	174	2420.2 (1919.6–3051.3)	187	3465.7 (2858.7–4201.6)	170	3283.0 (2664.3–4045.4)
9V	185	1651.1 (1352.0–2016.4)	174	1809.2 (1502.2–2179.0)	187	1730.3 (1432.7–2089.8)	166	2026.6 (1651.0–2487.5)
14	184	3048.3 (2429.0–3825.5)	176	2618.4 (2078.8–3298.1)	186	2487.2 (2026.1–3053.2)	177	2460.8 (2018.4–3000.3)
18C	188	1592.9 (1281.0–1980.7)	179	2033.7 (1639.6–2522.5)	187	3144.5 (2597.3–3806.8)	171	3183.5 (2587.9–3916.2)
19A	185	2135.7 (1761.7–2589.2)	169	2626.1 (2139.6–3223.2)	173	3214.3 (2735.5–3776.9)	162	4037.4 (3234.4–5039.8)
19F	180	1379.9 (1144.4–1663.9)	167	1532.6 (1250.5–1878.5)	182	1742.0 (1440.1–2107.2)	164	1950.1 (1592.9–2387.4)
22F	175	2023.0 (1559.4–2624.5)	170	2178.4 (1630.9–2909.9)	175	2282.7 (1804.7–2887.3)	156	2670.6 (2062.6–3457.9)
23F	183	739.0 (559.4–976.3)	169	839.9 (639.5–1103.1)	181	2326.3 (1831.2–2955.2)	169	2212.0 (1685.3–2903.3)
33F	176	10,089.0 (7799.0–13,051.4)	166	10,909.2 (8634.0–13,783.9)	175	6788.5 (5312.9–8673.9)	166	9339.7 (7330.9–11,899.0)

^†^ The within-group 95% CIs are obtained by exponentiating the CIs of the mean of the natural log values based on the t-distribution. n = number of participants contributing to the analysis. Note: Post-vaccination = 30 days following vaccination with PPSV23 or PCV15 (Day 30 for PPSV23 or PCV15 concomitant groups and Day 60 for PPSV23 or PCV15 sequential groups). CI: confidence interval; OPA: opsonophagocytic activity; PCV15: 15-valent pneumococcal conjugate vaccine; PPSV23: 23-valent pneumococcal polysaccharide vaccine.

**Table 3:** Primary immunogenicity endpoint: summary of SARS-CoV-2-specific binding antibody GMTs responses with mRNA-1273.

	n	Observed GMT Response (Baseline), 1/dil (95% CI)	n	Observed GMT Response, 1/dil (95% CI)	n	GMFR	n	% ≥4-Fold Rise
PPSV23 concomitant group (N = 214)	203	96,219.3 (74,340.0–124,538.0)	197	824,118.4 (717,446.4–946,650.6)	196	8.6 (6.7–11.0)	196	55.1 (47.9–62.2)
PCV15 concomitant group (N = 210)	201	104,885.7 (80,862.2–136,046.3)	196	861,746.4 (756,496.4–981,639.8)	195	8.1 (6.4–10.2)	195	52.3 (45.1–59.5)
PPSV23 and PCV15 sequential groups (N = 419)	403	115,174.3 (96,328.9–137,706.4)	382	1,102,295.7 (1,003,694.1–1,210,583.7)	379	9.1 (7.7–10.8)	379	55.9 (50.8–61.0)

CI: confidence interval; GMFR: geometric mean fold rise; GMT: geometric mean titer; N: number of participants randomized and vaccinated; n: number of participants contributing to the analysis; PCV15: 15-valent pneumococcal conjugate vaccine; PPSV23: 23-valent pneumococcal polysaccharide vaccine; SARS-CoV-2: severe acute respiratory syndrome coronavirus 2.

**Table 4:** Safety summary.

Participants in Population, n (%)	PPSV23 Concomitant Group (n = 214)	PPSV23 Sequential Group (n = 211)	PCV15 Concomitant Group (n = 209)	PCV15 Sequential Group (n = 208)
**At least one AE**	175 (81.8)	171 (81.0)	164 (78.5)	174 (83.7)
Injection-site	164 (76.6)	162 (76.8)	154 (73.7)	164 (78.8)
Systemic	132 (61.7)	123 (58.3)	121 (57.9)	132 (63.5)
**No AEs reported**	39 (18.2)	40 (19.0)	45 (21.5)	34 (16.3)
**Vaccine-related AEs ^†^**	171 (79.9)	170 (80.6)	164 (78.5)	172 (82.7)
Injection-site	164 (76.6)	162 (76.8)	154 (73.7)	164 (78.8)
Systemic	119 (55.6)	108 (51.2)	111 (53.1)	124 (59.6)
**At least one solicited AE**	171 (79.9)	170 (80.6)	163 (78.0)	171 (82.2)
**At least one systemic AE**				
Following vaccination 1	124 (57.9)	227 (54.2)	111 (53.1)	227 (54.2)
Following vaccination 2	85 (20.7)	51 (25.1)	85 (20.7)	78 (39.2)
**At least one systemic vaccine-related AE ^†^**	119 (55.6)	108 (51.2)	111 (53.1)	124 (59.6)
**SAEs**	2 (0.9)	2 (0.9)	6 (2.9)	4 (1.9)
**Serious vaccine-related AEs**	0 (0.0)	0 (0.0)	0 (0.0)	0 (0.0)
**Deaths**	0 (0.0)	0 (0.0)	0 (0.0)	1 (0.5)
**AEs leading to discontinuation**	1 (0.5)	0 (0.0)	0 (0.0)	2 (1.0)
**Vaccine-related AEs leading to discontinuation**	1 (0.5)	0 (0.0)	0 (0.0)	0 (0.0)
**SAEs leading to discontinuation**	0 (0.0)	0 (0.0)	0 (0.0)	2 (1.0)
**Serious vaccine-related AEs leading to discontinuation**	0 (0.0)	0 (0.0)	0 (0.0)	0 (0.0)

^†^ Vaccine-related systemic AEs cannot be attributed to a specific vaccine following vaccination 1. MedDRA version 26.0 was used. AE: adverse event; MedDRA: Medical Dictionary for Regulatory Activities; PCV15: 15-valent pneumococcal conjugate vaccine; PPSV23: 23-valent pneumococcal polysaccharide vaccine; SAE: serious adverse event.

## Data Availability

The data sharing policy, including restrictions, of Merck Sharp & Dohme LLC, a subsidiary of Merck & Co., Inc., Rahway, NJ, USA (MSD), is available at https://trialstransparency.msdclinicaltrials.com/policies-perspectives.aspx (accessed on 13 February 2025). Requests for access to the clinical study data can be submitted via email to the Data Access mailbox (mailto:dataaccess@msd.com).

## Supplementary Materials

The following supporting information can be downloaded at: https://www.mdpi.com/article/10.3390/vaccines13020192/s1, Table S1: Investigators. The following investigators randomized at least one participant in the clinical study; Table S2: Eligibility criteria; Table S3: Definition of adverse events of special interest; Table S4: Baseline characteristics and demographics by strata; Table S5: Pre-existing medical conditions occurring in ≥5% of participants (any group); Table S6: Secondary immunogenicity endpoint: Serotype-specific GMFRs and proportions of participants with ≥4-fold rise for OPA responses at 30 days post-vaccination with PPSV23; Table S7: Secondary immunogenicity endpoints: GMFRs and proportions of participants with ≥4-fold rise for OPA antibody responses at 30 days post-vaccination with PCV15; Table S8: Most frequently reported AEs (≥5% incidence in any group); Figure S1: Reverse cumulative distribution curve of OPA titers at baseline and 30 days post-vaccination in the PPSV23 groups; Figure S2: Reverse cumulative distribution curve of OPA titers at baseline and 30 days post-vaccination in the PCV15 groups; Figure S3: Reverse cumulative distribution curve of SARS-CoV-2-specific binding titers at baseline and 30 days post-vaccination with mRNA-1273.
