# Correction: Omole et al. Safety, Tolerability, and Immunogenicity of the Pneumococcal Vaccines PPSV23 or PCV15 Co-Administered with a Booster Dose of mRNA-1273 SARS-CoV-2 Vaccine in Healthy Adults ≥50 Years of Age. *Vaccines* 2025, *13*, 192

**DOI:** 10.3390/vaccines13101056

**Published:** 2025-10-16

**Authors:** Tosin Omole, Enrique Pelayo, Aaron S. Weinberg, Spyros Chalkias, Zelalem Endale, Gretchen Tamms, Tina M. Sterling, Lori Good, Tulin Shekar, Morgan Johnson, Natalie Banniettis, Ulrike K. Buchwald, Alejandra Esteves-Jaramillo

**Affiliations:** 1 Merck & Co., Inc., Rahway, NJ 07065, USA; gretchen_tamms@merck.com (G.T.); tina_sterling@merck.com (T.M.S.); lori_good@merck.com (L.G.); tulin.shekar@merck.com (T.S.); morgan.johnson@merck.com (M.J.); natalie.banniettis@merck.com (N.B.); ulrike.buchwald@merck.com (U.K.B.); alex.esteves@merck.com (A.E.-J.); 2 Advanced Medical Research Institute, Miami, FL 33174, USA; epelayomd@amriresearch.com; 3 Carbon Health, North Hollywood, CA 91606, USA; aaron@carbonhealth.com; 4 Moderna, Cambridge, MA 02142, USA; spyros.chalkias@modernatx.com (S.C.); zelalem.endale@modernatx.com (Z.E.)

The authors would like to make the following corrections to this published paper [1].

In the original publication, there was a typographical error in the footnote for Table 1. Information in footnotes indicating previous receipt of non-study vaccines in the PPSV23 and PCV15 groups was reversed. The footnote indicated with † has been changed from “A participant in the PPSV23 sequential group had previously received an inactivated vaccine for influenza” to “A participant in the PPSV23 sequential group had previously received a vaccine for tetanus”. The footnote indicated with ‡ has been changed from “A participant in the PCV15 group had previously received a vaccine for tetanus” to “A participant in the PCV15 group had previously received an inactivated vaccine for influenza”. The corrected Table 1 appears below.

In the original publication, there were typographical errors in the population sizes for the PCV15 concomitant and PCV15 sequential groups in Table 2. The population size for the PCV15 concomitant group has been changed from “n = 211” to “n = 210”, and population size for the PCV15 sequential group has been changed from “n = 211” to “n = 208”. The corrected Table 2 appears below.

The authors state that the scientific conclusions are unaffected. This correction was approved by the Academic Editor. The original publication has also been updated.

## Figures and Tables

**Table 1:** Baseline characteristics and demographics.

Participants in Population	PPSV23 Concomitant Group (n = 214)	PPSV23 Sequential Group (n = 211)	PCV15 Concomitant Group (n = 210)	PCV15 Sequential Group (n = 208)
**Sex, n (%)**				
Male	107 (50.0)	80 (37.9)	98 (46.7)	78 (37.5)
Female	107 (50.0)	131 (62.1)	112 (53.3)	130 (62.5)
**Mean age, years (range)**	60.5 (50–93)	60.5 (50–89)	60.5 (50–88)	60.4 (50–86)
**Age category**				
50–64 years	158 (73.8)	155 (73.5)	153 (72.9)	153 (73.6)
65–74 years	41 (19.2)	42 (19.9)	43 (20.5)	43 (20.7)
≥75 years	15 (7.0)	14 (6.6)	14 (6.7)	12 (5.8)
**Race, n (%)**				
White	164 (76.6)	173 (82.0)	163 (77.6)	157 (75.5)
Black or African American	41 (19.2)	32 (15.2)	36 (17.1)	40 (19.2)
Asian	4 (1.9)	3 (1.4)	7 (3.3)	4 (1.9)
Multiple	5 (2.3)	3 (1.4)	3 (1.4)	5 (2.4)
American Indian or Alaska Native	0	0	1 (0.5)	2 (1.0)
**Ethnicity, n (%)**				
Not Hispanic or Latino	127 (59.3)	111 (52.6)	122 (58.1)	116 (55.8)
Hispanic or Latino	87 (40.7)	98 (46.4)	88 (41.9)	91 (43.8)
Not reported	0	2 (0.9)	0	1 (0.5)
**Prior medications, n (%)**				
At least one prior medication	154 (72.0)	162 (76.8)	160 (76.2)	148 (71.2)
No prior medication	60 (28.0)	49 (23.2)	50 (23.8)	60 (28.8)
**Prior vaccinations, n (%)**				
At least one prior vaccination	0	1 (0.5) ^†^	1 (0.5) ^‡^	0
No prior vaccinations	214 (100.0)	210 (99.5)	209 (99.5)	208 (100.0)
**Concomitant medications, n (%)**				
At least one concomitant medication	164 (76.6)	173 (82.0)	167 (79.5)	162 (77.9)
No concomitant medications	50 (23.4)	38 (18.0)	43 (20.5)	46 (22.1)
**Concomitant vaccinations, n (%)**				
At least one concomitant vaccination	4 (1.9)	1 (0.5)	7 (3.3)	4 (1.9)
No concomitant vaccinations	210 (98.1)	210 (99.5)	203 (96.7)	204 (98.1)

Each participant is counted once per parameter. ^†^ A participant in the PPSV23 sequential group had previously received a vaccine for tetanus. ^‡^ A participant in the PCV15 group had previously received an inactivated vaccine for influenza. PCV15: 15-valent pneumococcal conjugate vaccine; PPSV23: 23-valent pneumococcal polysaccharide vaccine.

**Table 2:** Primary immunogenicity endpoint: serotype-specific OPA responses at 30 days post-vaccination.

	PPSV23 Concomitant Group (n = 214)		PPSV23 Sequential Group (n = 211)		PCV15 Concomitant Group (n = 210)		PCV15 Sequential Group (n = 208)	
Pneumococcal Serotype	n	Observed Response (95% CI) ^†^	n	Observed Response (95% CI) ^†^	n	Observed Response (95% CI) ^†^	n	Observed Response (95% CI) ^†^
1	182	236.0 (177.0–314.6)	177	253.5 (188.4–341.1)	186	208.3 (160.8–269.9)	174	265.6 (203.4–346.7)
3	167	174.3 (138.6–219.2)	164	284.0 (224.1–359.9)	174	237.5 (197.5–285.6)	155	338.4 (275.3–416.0)
4	182	1384.5 (1110.7–1725.9)	165	1704.4 (1370.6–2119.5)	181	1586.0 (1296.5–1940.2)	176	1758.6 (1419.4–2178.8)
5	189	423.4 (324.9–551.7)	181	376.3 (282.6–501.2)	190	447.0 (341.7–584.8)	180	505.5 (382.7–667.8)
6A	–	–	–	–	176	4345.3 (3490.0–5410.1)	162	5787.1 (4468.4–7495.0)
6B	182	1336.7 (1038.2–1721.1)	171	1326.8 (1016.7–1731.4)	173	3655.5 (2939.1–4546.6)	158	4992.9 (3896.2–6398.2)
7F	184	2614.4 (2123.8–3218.4)	174	2420.2 (1919.6–3051.3)	187	3465.7 (2858.7–4201.6)	170	3283.0 (2664.3–4045.4)
9V	185	1651.1 (1352.0–2016.4)	174	1809.2 (1502.2–2179.0)	187	1730.3 (1432.7–2089.8)	166	2026.6 (1651.0–2487.5)
14	184	3048.3 (2429.0–3825.5)	176	2618.4 (2078.8–3298.1)	186	2487.2 (2026.1–3053.2)	177	2460.8 (2018.4–3000.3)
18C	188	1592.9 (1281.0–1980.7)	179	2033.7 (1639.6–2522.5)	187	3144.5 (2597.3–3806.8)	171	3183.5 (2587.9–3916.2)
19A	185	2135.7 (1761.7–2589.2)	169	2626.1 (2139.6–3223.2)	173	3214.3 (2735.5–3776.9)	162	4037.4 (3234.4–5039.8)
19F	180	1379.9 (1144.4–1663.9)	167	1532.6 (1250.5–1878.5)	182	1742.0 (1440.1–2107.2)	164	1950.1 (1592.9–2387.4)
22F	175	2023.0 (1559.4–2624.5)	170	2178.4 (1630.9–2909.9)	175	2282.7 (1804.7–2887.3)	156	2670.6 (2062.6–3457.9)
23F	183	739.0 (559.4–976.3)	169	839.9 (639.5–1103.1)	181	2326.3 (1831.2–2955.2)	169	2212.0 (1685.3–2903.3)
33F	176	10,089.0 (7799.0–13,051.4)	166	10,909.2 (8634.0–13,783.9)	175	6788.5 (5312.9–8673.9)	166	9339.7 (7330.9–11,899.0)

^†^ The within-group 95% CIs are obtained by exponentiating the CIs of the mean of the natural log values based on the t-distribution. n = number of participants contributing to the analysis. Note: Post-vaccination = 30 days following vaccination with PPSV23 or PCV15 (Day 30 for PPSV23 or PCV15 concomitant groups and Day 60 for PPSV23 or PCV15 sequential groups). CI: confidence interval; OPA: opsonophagocytic activity; PCV15: 15-valent pneumococcal conjugate vaccine; PPSV23: 23-valent pneumococcal polysaccharide vaccine.
