# Supplementary material for: Safety, Tolerability, and Immunogenicity of the Pneumococcal Vaccines PPSV23 or PCV15 Co-Administered with a Booster Dose of mRNA-1273 SARS-CoV-2 Vaccine in Healthy Adults ≥50 Years of Age
Source: Vaccines (Basel). 2025 Feb 15;13(2):192. doi: 10.3390/vaccines13020192 (PMC11860627; doi:10.3390/vaccines13020192)
Supplement: Supplementary file 1 [file vaccines-13-00192-s001.zip › vaccines-3434398-supplementary.pdf]

Supplementary Table S1. Investigators.

The following investigators randomized at least one participant in the clinical study:

Country	Investigators
US	**Jose Francisco Cardona**, Indago Research and Health Center Inc, Hialeah, FL
	**Enrique Pelayo,** Advanced Medical Research, LLC, Miami, FL
	**Celia Reyes-Acuna,** South Texas Clinical Research, Corpus Christi, TX
	**Daniel Brune**, Optimal Research, Peoria, IL
	**Aaron Samuel Weinberg**, Carbon Health, San Francisco, CA
	**Anthony D. Puopolo,** Community Clinical Research Center, East Marlborough, MA
	**Masoud Mohammed Azizad**, Valley Clinical Trials Inc, Northridge, CA
	**Donald M. Brandon**, California Research Foundation, San Diego, CA
	**Charles P Andrews**, Diagnostics Research Group, San Antonio, TX
	**Alberto J. Odio**, Millennium Clinical Trials, Simi Valley, CA
	**Matthew Gilruth Davis**, Rochester Clinical Research, Inc., Rochester, NY
	**George Hartley Freeman**, Health Research of Hampton Roads, Inc, Newport News, VA,
	**Richard Holt Leggett**, Crossroads Clinical Research LLC, Victoria, TX
	**Benidecto Fernandez**, Lakes Research LLC, Miami Lakes, FL
	**Cynthia Becher Strout/Rica Santiago**, Coastal Carolina Research Center, North Charleston, SC
	**Helen L Stacey**, Diablo Clinical Research, Inc, Walnut Creek, CA
	**Zahid Zafar**, Wellness Clinical Research Associates, McKinney, TX
	**Robert A. Riesenberg**, Atlanta Center for Medical Research, Atlanta, GA
	**Christopher A. Smith**, Certified Research Associates, Cortland, NY
	**Edwin Mangune**, Center for Clinical Trials, LLC, Paramount, CA
	**Vicki Miller**, DM Clinical Research, Tomball, TX
	**Daniel Johnson**, Artemis Institute for Clinical Research, San Diego, CA
	**Michael Lewis Levin/Liliana I Ruiz-Leon**, Wake Research Clinical Research Center of Nevada, LLC, Las Vegas, NV

	**Barbara Claire Fleming-Phillips**, AMR Lexington, Lexington, KY<br><br>**Joan Rothenberg/Margaret Rhee**, Velocity Clinical Research-Cleveland, Beachwood, OH<br><br>**Barbara E. Rizzardi,** Velocity Clinical Research, West Jordan, UT<br><br>**Murray A. Kimmel**, Optimal Research LLC, Melbourne, FL<br><br>**Robert S. Call**, Clinical Research Partners, LLC., Richmond, VA<br><br>**Laurence Chu**, Benchmark Research, Austin, TX<br><br>**Steven A. Geller**, Centennial Medical Group, Elkridge, MD<br><br>**Brooke A. Dunlavy**, Alliance for Multispecialty Research, LLC, Newton, KS<br><br>**David L. Fried**, Velocity Clinical Research Providence, East Greenwich, RI<br><br>**John E. Ervin**, Alliance for Multispecialty Research, LLC, Kansas City, MO<br><br>**Mary C. L. Bailey**, Alliance for Multispecialty Research LLC (AMR – Norfolk), Norfolk, VA<br><br>**Jeffrey Bruce Rosen,** Alliance for Multispecialty Research, LLC, Coral Gables, FL<br><br>**Christopher A. Smith**, Corning Center for Clinical Research, Horseheads, NY<br><br>**William M. Seger**, Benchmark Research, Fort Worth, TX<br><br>**Megan Berman**, University of Texas Medical Branch at Galveston, Galveston, TX<br><br>**James R. Clark**, Charlottesville Medical Research Center, LLC, Charlottesville, VA<br><br>**Linda Gorgos**, AXCES Research Group, Santa Fe, NM
Puerto Rico	**Javier O. Morales-Ramirez,** Clinical Research Puerto Rico, San Juan, PR<br><br>**Elizabeth A. Barranco-Santana,** CAIMED Center – Ponce School of Medicine, Ponce, PR<br><br>**Evelyn Matta Fontanet**, Caparra Internal Medicine Research Center, Rio Grande, PR<br><br>**Ismael Toro-Grajales,** Cooperativa de Facultad Medica SANACOOP, Santa Cruz Bayamon, PR

Supplementary Table S2. Eligibility criteria.

Inclusion Criteria
**Type of Participant and Disease Characteristics**<br>• Is in good health. Any underlying chronic illness must be documented to be in stable condition<br>• Has received a two-dose regimen of the Moderna mRNA SARS-CoV-2 vaccine ≥5 months before enrollment<br>• In addition to the 2-dose primary series, a participant may have received either:<br>o A first booster dose of the Moderna mRNA SARS-CoV-2 vaccine ≥4 months (120 days) before receipt of study vaccine at first study visit, or<br>o No booster dose of the Moderna mRNA SARS-CoV-2 vaccine.<br><br>**Demographics**<br>• Is male or female, ≥50 years of age at the time of signing the informed consent<br><br>**Female Participants**<br>• A female participant is eligible to participate if she is not pregnant or breastfeeding, and at least one of the following conditions applies:<br>a. Is not a women of childbearing potential OR<br>b. Is a woman of childbearing potential and using an acceptable contraceptive method, or be abstinent from heterosexual intercourse as their preferred and usual lifestyle (abstinent on a long-term and persistent basis), as described in Appendix 5 during the intervention period and for at least 3 months after the last dose of study intervention. The investigator should evaluate the potential for contraceptive method failure (i.e., noncompliance, recently initiated) in relationship to the first dose of study intervention<br><br>**Additional Requirement(s)**<br>• The participant has the ability to complete electronic vaccination report card data collection without assistance
**Exclusion Criteria**
**Medical Conditions**<br>• Has a current SARS-CoV-2 infection or a known history of SARS-CoV-2 infection <3 months before receipt of study vaccine at first study visit<br>• Has a history of myocarditis and/or pericarditis<br>• Has a known hypersensitivity to any component of pneumococcal polysaccharide vaccine, PCV, any diphtheria toxoid-containing vaccine, or following a previous dose of pneumococcal polysaccharide vaccine or PCV<br>• Has a known hypersensitivity to any component of the mRNA-1273 vaccine or following a previous dose of any COVID-19 vaccine

- Has a known or suspected impairment of immunological function including, but not limited to, a history of congenital or acquired immunodeficiency, documented HIV infection, functional or anatomic asplenia, or history of autoimmune disease
- Has a coagulation disorder contraindicating intramuscular vaccinations
- *Had a recent febrile illness (defined as oral or tympanic temperature ≥100.4 °F [≥38.0 °C]; axillary or temporal temperature ≥99.4 °F [≥37.4 °C]) or received antibiotic therapy for an acute illness occurring <72 h before receipt of study vaccine
- Has a known malignancy that is progressing or has required active treatment <3 years before enrollment
    - Note: participants with basal cell carcinoma of the skin, squamous cell carcinoma of the skin, or carcinoma in situ (e.g., breast carcinoma, cervical cancer in situ) who have undergone potentially curative therapy are not excluded

Prior/Concomitant Therapy

- Received prior administration of a pneumococcal polysaccharide vaccine <5 years before study enrollment or is expected to receive a pneumococcal polysaccharide vaccine during the study outside the protocol
- Received prior administration of a PCV <1 year before study enrollment or is expected to receive a PCV during the study outside the protocol
- Received any SARS-CoV-2 vaccine other than the two-dose regimen of the Moderna mRNA vaccine, or is expected to receive any SARS-CoV-2 vaccine during the study outside the protocol
- Received systemic corticosteroids (≥20 mg/day prednisone equivalent) for ≥14 consecutive days and has not completed intervention ≥30 days before study enrollment
- Received systemic corticosteroids exceeding physiologic replacement doses (approximately 5 mg/day prednisone equivalent) ≤14 days before receipt of study vaccine
    - Note: Topical, ophthalmic, intraarticular, or soft-tissue (e.g., bursa, tendon steroid injections), and inhaled/nebulized steroids are permitted
- Is currently receiving immunosuppressive therapy, including chemotherapeutic agents used to treat cancer or other conditions, and interventions associated with organ or bone marrow transplantation, or autoimmune disease
- *Received any non-live vaccine ≤14 days before receipt of study vaccine or is scheduled to receive any non-live vaccine ≤30 days after receipt of study vaccine. Exception: Inactivated influenza vaccine allowed if given ≥7 days before or ≥15 days after receipt of study vaccine
- *Received any live virus vaccine ≤30 days before receipt of study vaccine or is scheduled to receive any live virus vaccine ≤30 days after receipt of study vaccine
- Received a blood transfusion or blood products (including globulin) ≤6 months before receipt of study vaccine or is scheduled to receive a blood transfusion or blood product ≤30 days after receipt of study vaccine. Autologous blood transfusions are not considered an exclusion criterion

Prior/Concurrent Clinical Study Experience

- Is currently participating in, or has participated in, an interventional clinical study with an investigational compound or device within 2 months of participating in this current study
- Received prior monoclonal antibody treatment for SARS-CoV-2 infection or received antiviral treatment for SARS-CoV-2 infection <3 months before receipt of study vaccine at first study visit

**Other Exclusions**
• In the opinion of the investigator, has a history of clinically relevant drug or alcohol use that would interfere with participation in protocol-specified activities<br>• Has a history or current evidence of any condition, therapy laboratory abnormality, or other circumstance that might predispose the participant to risk by participating in the study, confound the results of the study, or interfere with the participant's participation for the full duration of the study in the opinion of the investigator<br>• Is, or has, an immediate family member (e.g., spouse, parent/legal guardian, sibling, or child) who is investigational site or Sponsor staff directly involved with this study.

For items with an asterisk (*), if the participant meets these exclusion criteria, the Day 1 visit may be rescheduled for a time when these criteria are not met.
COVID-19: coronavirus disease 2019; h: hours; HIV: human immunodeficiency virus; PCV: pneumococcal conjugate vaccine; SARS-CoV-2: severe acute respiratory syndrome coronavirus 2.

Supplementary Table S3. Definition of adverse events of special interest.

Adverse Event	Definition
Anosmia, ageusia	New onset COVID-associated or idiopathic events without other etiology excluding congenital etiologies or trauma
Subacute thyroiditis	Including, but not limited to, events of atrophic thyroiditis, autoimmune thyroiditis, immune-mediated thyroiditis, silent thyroiditis, thyrotoxicosis, and thyroiditis
Acute pancreatitis	Including, but not limited to, events of autoimmune pancreatitis, immune-mediated pancreatitis, ischemic pancreatitis, edematous pancreatitis, pancreatitis, acute pancreatitis, hemorrhagic pancreatitis, necrotizing pancreatitis, viral pancreatitis, and subacute pancreatitis<br>Excluding known etiologic causes of pancreatitis (alcohol, gallstones, trauma, recent invasive procedures)
Appendicitis	Include any event of appendicitis
Rhabdomyolysis	New onset rhabdomyolysis without known etiology, such as excessive exercise or trauma
Acute respiratory distress syndrome	Including, but not limited to, new events of ARDS and respiratory failure
Coagulation disorders	Including, but not limited to, thromboembolic and bleeding disorders, disseminated intravascular coagulation, pulmonary embolism, deep vein thrombosis
Acute cardiovascular injury	Including, but not limited to, myocarditis, pericarditis, microangiopathy, coronary artery disease, arrhythmia, stress cardiomyopathy, heart failure, or acute myocardial infarction<br>In addition, investigators will be asked to report events of myocarditis and/or pericarditis as SAEs, and specifically as related if within 7 days of mRNA-1273 vaccination
Acute kidney injury	Including events with idiopathic or autoimmune etiologies<br>Excluding events with clear alternate etiology (trauma, infection, tumor, or iatrogenic causes, such as medications or radiocontrast agents, etc.)<br>Including all cases that meet the following criteria:<br>Increase in serum creatinine by ≥0.3 mg/dL (≥26.5 μmol/L) within 48 h OR increase in serum creatinine to ≥1.5 times baseline (based on history), known or presumed to have occurred within prior 7 days OR urine volume ≤0.5 mL/kg/h for 6 h
Acute liver injury	Including events with idiopathic or autoimmune etiologies. Excluding events with clear alternate etiology (trauma, infection, tumor, etc.)

	Including all cases that meet the following criteria: >3-fold elevation above the upper normal limit for ALT or AST OR >2-fold elevation above the upper normal limit for total serum bilirubin or gamma glutamyl transferase or alkaline phosphatase
Dermatologic findings	• Chilblain-like lesions<br>• Single organ cutaneous vasculitis<br>• Erythema multiforme<br>• Bullous rashes<br>• Severe cutaneous adverse reactions including, but not limited to: Stevens-Johnson syndrome, toxic epidermal necrolysis, drug reaction with eosinophilia and systemic symptoms, and fixed drug eruptions
Multisystem inflammatory disorders	• Multisystem inflammatory syndrome in adults<br>• Kawasaki's disease
Thrombocytopenia	Platelet counts <150 × 109 per mm^3<br>Including, but not limited to, immune thrombocytopenia, platelet production decreased, thrombocytopenia, thrombocytopenic purpura, thrombotic thrombocytopenic purpura, or hemolysis, elevated liver enzymes, low platelet count (HELLP) syndrome
Acute aseptic arthritis	New onset aseptic arthritis without clear alternate etiology (e.g., gout, osteoarthritis, and trauma)
New onset of or worsening of neurologic disease	Including but not limited to:<br>• Guillain–Barre syndrome<br>• Acute disseminated encephalomyelitis<br>• Peripheral facial nerve palsy (Bell's palsy)<br>• Transverse myelitis<br>• Encephalitis/encephalomyelitis<br>• Aseptic meningitis<br>• Febrile seizures<br>• Generalized seizures/convulsions<br>• Stroke (hemorrhagic and non-hemorrhagic)<br>• Narcolepsy
Anaphylaxis	Anaphylaxis is an acute hypersensitivity reaction with multi-organ system involvement that can present as, or rapidly progress to, a severe life-threatening reaction. It may occur following exposure to allergens from a variety of sources<br>Anaphylaxis is a clinical syndrome characterized by the following:<br>• Sudden onset AND<br>• Rapid progression of signs and symptoms AND

	• Involves two or more organ systems, as follows:<br>**Skin/mucosal:** urticaria (hives), generalized erythema, angioedema, generalized pruritus with skin rash, generalized prickle sensation, and red and itchy eyes<br>**Cardiovascular:** measured hypotension, clinical diagnosis of uncompensated shock, loss of consciousness or decreased level of consciousness, and evidence of reduced peripheral circulation<br>**Respiratory:** bilateral wheeze (bronchospasm), difficulty breathing, stridor, upper airway swelling (lip, tongue, throat, uvula, or larynx), respiratory distress, persistent dry cough, hoarse voice, sensation of throat closure, sneezing, and rhinorrhea<br>**Gastrointestinal:** diarrhea, abdominal pain, nausea, and vomiting
Other syndromes	• Fibromyalgia<br>• Postural orthostatic tachycardia syndrome<br>• Chronic fatigue syndrome (includes myalgic encephalomyelitis and post-viral fatigue syndrome)<br>• Myasthenia gravis

ALT: alanine aminotransferase; ARDS: acute respiratory distress syndrome; AST: aspartate aminotransferase; COVID: coronavirus disease; HELLP: hemolysis, elevated liver enzymes, low platelet count; SAE: serious adverse event.

Supplementary Table S4. Baseline characteristics and demographics by strata.

**Participants in Population, n (%)**	**PPSV23 Concomitant Group (n = 214)**	**PPSV23 Sequential Group (n = 211)**	**PCV15 Concomitant Group (n = 210)**	**PCV15 Sequential Group (n = 208)**
50–64 years of age + prior history of pneumococcal vaccination	12 (5.6)	12 (5.7)	12 (5.7)	12 (5.8)
50–64 years of age + pneumococcal vaccine naive	141 (65.9)	140 (66.4)	137 (65.2)	139 (66.8)
65–74 years of age + prior history of pneumococcal vaccination	8 (3.7)	9 (4.3)	8 (3.8)	8 (3.8)
65–74 years of age + pneumococcal vaccine naive	33 (15.4)	33 (15.6)	34 (16.2)	33 (15.9)
≥75 years of age + prior history of pneumococcal vaccination	5 (2.3)	5 (2.4)	5 (2.4)	4 (1.9)
≥75 years of age + pneumococcal vaccine naive	9 (4.2)	9 (4.3)	9 (4.3)	9 (4.3)
50–64 years of age + pneumococcal vaccine naïve + no prior booster + no prior SARS-CoV-2 infection	1 (0.5)	1 (0.5)	1 (0.5)	1 (0.5)
65–74 years of age + pneumococcal vaccine naïve + no prior booster + no prior SARS-CoV-2 infection	0	0	1 (0.5)	0
50–64 years of age + pneumococcal vaccine naïve + history of booster + no prior SARS-CoV-2 infection	3 (1.4)	2 (0.9)	3 (1.4)	2 (1.0)
65–74 years of age + pneumococcal vaccine naïve + history of booster + no prior SARS-CoV-2 infection	1 (0.5)	0	0	0
≥75 years of age + pneumococcal vaccine naïve + history of booster + no prior SARS-CoV-2 infection	1 (0.5)	0	0	0

PCV15: 15-valent pneumococcal conjugate vaccine; PPSV23: 23-valent pneumococcal polysaccharide vaccine; SARS-CoV-2: severe acute respiratory syndrome coronavirus 2.

Supplementary Table S5. Pre-existing medical conditions occurring in ≥5% of participants (any group)

Pre-existing condition, n (%)	PPSV23 Concomitant Group (n = 214)	PPSV23 Sequential Group (n = 211)	PCV15 Concomitant Group (n = 210)	PCV15 Sequential Group (n = 208)
At least one medical condition	181 (84.6)	185 (87.7)	191 (91.0)	181 (87.0)
No medical conditions	33 (15.4)	26 (12.3)	19 (9.0)	27 (13.0)
Hypertension	81 (37.9)	80 (37.9)	76 (36.2)	71 (34.1)
Type 2 diabetes mellitus	38 (17.8)	45 (21.3)	37 (17.6)	28 (13.5)
Postmenopausal	30 (14.0)	38 (18.0)	40 (19.0)	28 (13.5)
Hypothyroidism	30 (14.0)	23 (10.9)	18 (8.6)	17 (8.2)
Obesity	26 (12.1)	28 (13.3)	29 (13.8)	29 (13.9)
Hyperlipidemia	25 (11.7)	19 (9.0)	17 (8.1)	22 (10.6)
Gastroesophageal reflux disease	24 (11.2)	37 (17.5)	26 (12.4)	35 (16.8)
Depression	23 (10.7)	32 (15.2)	20 (9.5)	36 (17.3)
Hypercholesterolemia	20 (9.3)	32 (15.2)	17 (8.1)	12 (5.8)
Osteoarthritis	18 (8.4)	30 (14.2)	27 (12.9)	29 (13.9)
Drug hypersensitivity	18 (8.4)	21 (10.0)	24 (11.4)	28 (13.5)
Seasonal allergy	16 (7.5)	20 (9.5)	18 (8.6)	17 (8.2)
Tobacco user	14 (6.5)	10 (4.7)	18 (8.6)	17 (8.2)
Anxiety	13 (6.1)	22 (10.4)	17 (8.1)	29 (13.9)
Asthma	13 (6.1)	14 (6.6)	17 (8.1)	23 (11.1)
Insomnia	11 (5.1)	25 (11.8)	17 (8.1)	20 (9.6)
Back pain	11 (5.1)	12 (5.7)	18 (8.6)	18 (8.7)
Overweight	9 (4.2)	16 (7.6)	13 (6.2)	13 (6.3)
Allergic rhinitis	8 (3.7)	12 (5.7)	11 (5.2)	6 (2.9)
Essential hypertension	6 (2.8)	17 (8.1)	13 (6.2)	15 (7.2)
Hysterectomy	5 (2.3)	16 (7.6)	8 (3.8)	17 (8.2)

Dyslipidemia	5 (2.3)	14 (6.6)	9 (4.3)	9 (4.3)
Elevated blood cholesterol	5 (2.3)	7 (3.3)	8 (3.8)	13 (6.3)
Myopia	3 (1.4)	4 (1.9)	11 (5.2)	7 (3.4)

Each participant is only counted once per medical condition.
PCV15: 15-valent pneumococcal conjugate vaccine; PPSV23: 23-valent pneumococcal polysaccharide vaccine.

Supplementary Table S6. Secondary immunogenicity endpoint: Serotype-specific GMFRs and proportions of participants with ≥4-fold rise for OPA responses at 30 days post-vaccination with PPSV23.

Pneumococcal Serotype	Endpoint	PPSV23 Concomitant Group (n = 214)		PPSV23 Sequential Group (n = 211)	
		n	Observed Response (95% CI)	n	Observed Response (95% CI)
1	GMT (baseline)	193	10.2 (8.4, 12.5)	190	11.1 (8.9, 13.8)
	GMT (post-vaccination)	182	236.0 (177.0, 314.6)	177	253.5 (188.4, 341.1)
	GMFR	175	24.8 (18.3, 33.7)	173	24.2 (17.8, 33.0)
	% ≥4-fold rise	175	76.0% (133/175) (69.0, 82.1)	173	75.1% (130/173) (68.0, 81.4)
3	GMT (baseline)	180	25.2 (20.9, 30.3)	182	24.5 (20.4, 29.5)
	GMT (post-vaccination)	167	174.3 (138.6, 219.2)	164	284.0 (224.1, 359.9)
	GMFR	151	7.1 (5.7, 8.9)	153	11.6 (9.1, 14.8)
	% ≥4-fold rise	151	66.9% (101/151) (58.8, 74.3)	153	72.5% (111/153) (64.8, 79.4)
4	GMT (baseline)	188	99.0 (76.2, 128.6)	181	95.2 (73.2, 123.9)
	GMT (post-vaccination)	182	1384.5 (1110.7, 1725.9)	165	1704.4 (1370.6, 2119.5)
	GMFR	169	12.8 (9.5, 17.3)	154	15.7 (11.5, 21.3)
	% ≥4-fold rise	169	66.3% (112/169) (58.6/73.4)	154	68.2% (105/154) (60.2, 75.4)
5	GMT (baseline)	192	27.8 (22.9, 33.8)	189	26.7 (22.1, 32.3)
	GMT (post-vaccination)	189	423.4 (324.9, 551.7)	181	376.3 (282.6, 501.2)
	GMFR	180	15.1 (11.5, 19.9)	176	13.8 (10.4, 18.2)
	% ≥4-fold rise	180	75.0% (135/180) (68.0, 81.1)	176	69.9% (123/176) (62.5, 76.6)

6B	GMT (baseline)	190	177.9 (133.2, 237.7)	186	164.7 (124.3, 218.2)
	GMT (post-vaccination)	182	1336.7 (1038.2, 1721.1)	171	1326.8 (1016.7, 1731.4)
	GMFR	172	8.2 (6.0, 11.2)	164	8.6 (6.3, 11.6)
	% ≥4-fold rise	172	55.8% (96/172) (48.1, 63.4)	164	56.7% (93/164) (48.8, 64.4)
7F	GMT (baseline)	186	180.9 (138.7, 235.9)	180	164.7 (125.1, 216.8)
	GMT (post-vaccination)	184	2614.4 (2123.8, 3218.4)	174	2420.2 (1919.6, 3051.3)
	GMFR	170	14.5 (11.0, 19.2)	162	14.8 (10.8, 20.2)
	% ≥4-fold rise	170	71.2% (121/170) (63.7, 77.9)	162	66.7% (108/162) (58.8, 73.9)
9V	GMT (baseline)	185	403.2 (327.7, 496.2)	178	350.1 (286.9, 427.3)
	GMT (post-vaccination)	185	1651.1 (2429.0, 3825.5)	174	1809.2 (1502.2, 2179.0)
	GMFR	170	4.3 (3.4, 5.4)	160	5.3 (4.2, 6.7)
	% ≥4-fold rise	170	47.1% (80/170) (39.4, 54.9)	160	51.9% (83/160) (43.8, 59.8)
14	GMT (baseline)	186	496.1 (383.4, 642.0)	185	382.4 (292.3, 500.3)
	GMT (post-vaccination)	184	3048.3 (2429.0, 3825.5)	176	2618.4 (2078.8, 3298.1)
	GMFR	168	6.0 (4.6, 7.7)	168	7.0 (5.3, 9.2)
	% ≥4-fold rise	168	49.4% (83/168) (41.6, 57.2)	168	52.4% (88/168) (44.5, 60.1)
18C	GMT (baseline)	193	160.0 (133.1, 192.2)	187	182.8 (149.5, 223.5)
	GMT (post-vaccination)	188	1592.9 (1281.0, 1980.7)	179	2033.7 (1639.6, 2522.5)
	GMFR	180	10.2 (8.1, 12.9)	173	10.9 (8.5, 13.9)
	% ≥4-fold rise	180	66.7% (120/180) (59.3, 73.5)	173	69.9% (121/173) (62.5, 76.7)
19A	GMT (baseline)	191	288.1 (222.3, 373.6)	183	334.2 (260.4, 428.8)

	GMT (post-vaccination)	185	2135.7 (1761.7, 2589.2)	169	2626.1 (2139.6, 3223.2)
	GMFR	174	7.8 (6.1, 9.9)	161	8.7 (6.7, 11.3)
	% ≥4-fold rise	174	61.5% (107/174) (53.8, 68.8)	161	62.1% (100/161) (54.1, 69.6)
19F	GMT (baseline)	179	213.9 (175.5, 260.8)	178	222.7 (183.5, 270.4)
	GMT (post-vaccination)	180	1379.9 (1144.4, 1663.9)	167	1532.6 (1250.5, 1878.5)
	GMFR	162	6.9 (5.3, 8.8)	156	6.7 (5.4, 8.4)
	% ≥4-fold rise	162	62.3% (101/162) (54.4, 69.8)	156	60.3% (94/156) (52.1, 68.0)
22F	GMT (baseline)	166	74.2 (51.2, 107.4)	169	70.2 (48.3, 102.1)
	GMT (post-vaccination)	175	2023.0 (1559.4, 2624.5)	170	2178.4 (1630.9, 2909.9)
	GMFR	146	25.6 (16.8, 39.0)	150	30.8 (20.1, 47.1)
	% ≥4-fold rise	146	68.5% (100/146) (60.3, 75.9)	150	66.0% (99/150) (57.8, 73.5)
23F	GMT (baseline)	182	145.6 (110.7, 191.4)	173	129.6 (98.5, 170.4)
	GMT (post-vaccination)	183	739.0 (559.4, 976.3)	169	839.9 (639.5, 1103.1)
	GMFR	167	4.7 (3.5, 6.5)	154	6.2 (4.6, 8.5)
	% ≥4-fold rise	167	44.3% (74/167) (36.6, 52.2)	154	53.9% (83/154) (45.7, 61.9)
33F	GMT (baseline)	186	896.8 (667.4, 1205.1)	184	745.7 (541.1, 1027.9)
	GMT (post-vaccination)	176	10,089.0 (7799.0, 13,051.4)	166	10,909.2 (8634.0, 13,783.9)
	GMFR	165	12.2 (8.9, 16.7)	156	16.9 (11.7, 24.3)
	% ≥4-fold rise	165	67.3% (111/165) (59.5, 74.4)	156	67.9% (106/156) (60.0, 75.2)

CI: confidence interval; GMFR: geometric mean fold rise; GMT: geometric mean titer; OPA: opsonophagocytic activity; PPSV23: 23-valent pneumococcal polysaccharide vaccine.

Supplementary Table S7. Secondary immunogenicity endpoints: GMFRs and proportions of participants with ≥4-fold rise for OPA antibody responses at 30 days post-vaccination with PCV15.

Pneumococcal Serotype	Endpoint	PCV15 Concomitant Group (n = 210)		PCV15 Sequential Group (n = 208)	
		n	Observed Response (95% CI)	n	Observed Response (95% CI)
1	GMT (baseline)	192	12.6 (10.0, 15.8)	189	11.2 (9.0, 13.9)
	GMT (post-vaccination)	186	208.3 (160.8, 269.9)	174	265.6 (203.4, 346.7)
	GMFR	176	17.3 (12.8, 23.5)	173	24.5 (17.8, 33.6)
	% ≥4-fold rise	176	72.7% (128/176) (65.5, 79.2)	173	73.4% (127/173) (66.2, 79.8)
3	GMT (baseline)	186	26.1 (21.6, 31.5)	181	22.5 (18.5, 27.4)
	GMT (post-vaccination)	174	237.5 (197.5, 285.6)	155	338.4 (275.3, 416.0)
	GMFR	161	9.0 (7.0, 11.5)	150	14.5 (11.2, 18.8)
	% ≥4-fold rise	161	65.2% (105/161) (57.3, 72.5)	150	79.3% (119/150) (72.0, 85.5)
4	GMT (baseline)	184	124.2 (94.5, 163.3)	176	83.0 (62.7, 109.9)
	GMT (post-vaccination)	181	1586.0 (1296.5, 1940.2)	176	1758.6 (1419.4, 2178.8)
	GMFR	164	13.3 (9.4, 18.6)	164	20.6 (14.8, 28.8)
	% ≥4-fold rise	164	68.9% (113/164) (61.2, 75.9)	164	73.2% (120/164) (65.7, 79.8)
5	GMT (baseline)	194	30.9 (24.8, 38.4)	189	26.9 (22.1, 32.7)
	GMT (post-vaccination)	190	447.0 (341.7, 584.8)	180	505.5 (382.7, 667.8)
	GMFR	183	14.7 (11.2, 19.4)	179	18.7 (13.9, 25.2)
	% ≥4-fold rise	183	70.5% (129/183) (63.3, 77.0)	179	73.2% (131/179) (66.1, 79.5)
6A	GMT (baseline)	187	354.7 (292.1, 430.8)	173	276.8 (230.6, 332.1)
	GMT (post-vaccination)	176	4345.3 (3490.0, 5410.1)	162	5787.1 (4468.4, 7495.0)

	GMFR	162	12.7 (9.9, 16.4)	149	23.0 (17.0, 31.0)
	% ≥4-fold rise	162	75.9% (123/162) (68.6, 82.3)	149	82.6% (123/149) (75.5, 88.3)
6B	GMT (baseline)	193	193.6 (145.1, 258.2)	183	156.8 (117.8, 208.7)
	GMT (post-vaccination)	173	3655.5 (2939.1, 4546.6)	158	4992.9 (3896.2, 6398.2)
	GMFR	164	19.6 (14.0, 27.3)	153	28.4 (19.8, 40.9)
	% ≥4-fold rise	164	75.6% (124/164) (68.3, 82.0)	153	76.5% (117/153) (68.9, 82.9)
7F	GMT (baseline)	191	197.6 (150.4, 259.7)	184	202.3 (152.0, 269.3)
	GMT (post-vaccination)	187	3465.7 (2858.7, 4201.6)	170	3283.0 (2664.3, 4045.4)
	GMFR	177	17.6 (12.7, 24.3)	164	14.9 (10.8, 20.4)
	% ≥4-fold rise	177	67.8% (120/177) (60.4, 74.6)	164	65.2% (107/164) (57.4, 72.5)
9V	GMT (baseline)	190	378.4 (308.5, 464.0)	180	403.9 (326.2, 500.1)
	GMT (post-vaccination)	187	1730.3 (1432.7, 2089.8)	166	2026.6 (1651.0, 2487.5)
	GMFR	175	4.7 (3.7, 5.9)	158	5.2 (4.1, 6.5)
	% ≥4-fold rise	175	48.6% (85/175) (41.0, 56.2)	158	53.2% (84/158) (45.1, 61.1)
14	GMT (baseline)	194	558.8 (438.2, 712.6)	179	428.7 (326.6, 562.8)
	GMT (post-vaccination)	186	2487.2 (2026.1, 3053.2)	177	2460.8 (2018.4, 3000.3)
	GMFR	178	4.8 (3.6, 6.4)	166	6.3 (4.7, 8.3)
	% ≥4-fold rise	178	45.5% (81/178) (38.0, 53.1)	166	51.8% (86/166) (43.9, 59.6)
18C	GMT (baseline)	192	188.9 (152.8, 233.7)	185	184.5 (151.5, 224.7)
	GMT (post-vaccination)	187	3144.5 (2597.3, 3806.8)	171	3183.5 (2587.9, 3916.2)
	GMFR	177	16.6 (12.4, 22.3)	167	17.6 (13.5, 22.9)
	% ≥4-fold rise	177	72.9% (129/177) (65.7, 79.3)	167	75.4% (126/167) (68.2, 81.8)
19A	GMT (baseline)	190	339.7 (267.8, 431.1)	180	285.5 (215.5, 378.3)
	GMT (post-vaccination)	173	3214.3 (2735.5, 3776.9)	162	4037.4 (3234.4, 5039.8)

	GMFR	162	9.5 (7.1, 12.5)	156	15.2 (11.2, 20.7)
	% ≥4-fold rise	162	63.6% (103/162) (55.7, 71.0)	156	68.6% (107/156) (60.7, 75.8)
19F	GMT (baseline)	187	206.2 (170.2, 249.9)	177	207.1 (167.2, 256.5)
	GMT (post-vaccination)	182	1742.0 (1440.1, 2107.2)	164	1950.1 (1592.9, 2387.4)
	GMFR	168	8.6 (6.6, 11.2)	155	9.8 (7.5, 12.8)
	% ≥4-fold rise	168	66.1% (111/168) (58.4, 73.2)	155	67.7% (105/155) (59.8, 75.0)
22F	GMT (baseline)	179	59.8 (41.8, 85.4)	172	56.3 (39.0, 81.3)
	GMT (post-vaccination)	175	2282.7 (1804.7, 2887.3)	156	2670.6 (2062.6, 3457.9)
	GMFR	152	40.7 (25.7, 64.6)	142	43.9 (27.4, 70.2)
	% ≥4-fold rise	152	69.1% (105/152) (61.1, 76.3)	142	72.5% (103/142) (64.4, 79.7)
23F	GMT (baseline)	182	177.8 (133.7, 236.6)	174	118.8 (89.5, 157.7)
	GMT (post-vaccination)	181	2326.3 (1831.2, 2955.2)	169	2212.0 (1685.3, 2903.3)
	GMFR	163	13.9 (10.1, 19.1)	154	17.0 (12.1, 23.9)
	% ≥4-fold rise	163	65.0% (106/163) (57.2, 72.3)	154	70.1% (108/154) (62.2, 77.2)
33F	GMT (baseline)	188	926.8 (695.6, 1235.0)	181	833.5 (616.6, 1126.7)
	GMT (post-vaccination)	175	6788.5 (5312.9, 8673.9)	166	9339.7 (7330.9, 11,899.0)
	GMFR	162	7.9 (5.8, 10.9)	162	12.4 (8.7, 17.7)
	% ≥4-fold rise	162	56.2% (91/162) (48.2, 63.9)	162	63.6% (103/162) (55.7, 71.0)

CI: confidence interval; GMFR: geometric mean fold rise; GMT: geometric mean titer; OPA: opsonophagocytic activity; PCV15: 15-valent pneumococcal conjugate vaccine.

Supplementary Table S8. Most frequently reported AEs (≥5% incidence in any group).

Participants, n (%)	PPSV23 Concomitant and Sequential Groups Combined	PCV15 Concomitant and Sequential Groups Combined
Injection-site pain	317 (74.6)	308 (73.9)
Fatigue	166 (39.1)	185 (44.4)
Headache	137 (32.2)	141 (33.8)
Injection-site swelling	106 (24.9)	104 (24.9)
Myalgia	94 (22.1)	101 (24.2)
Lymphadenopathy	90 (21.2)	90 (21.6)
Chills	77 (18.1)	79 (18.9)
Injection-site erythema	71 (16.7)	68 (16.3)
Arthralgia	70 (16.5)	75 (18.0)
Nausea	51 (12.0)	54 (12.9)

Each participant is counted only once. Reported AEs include non-serious AEs within 28 days of any vaccination and serious AEs occurring throughout the duration of the study.
AE: adverse event; PCV15: 15-valent pneumococcal conjugate vaccine; PPSV23: 23-valent pneumococcal polysaccharide vaccine.

Supplementary Figure S1. Reverse cumulative distribution curve of OPA titers at baseline and 30 days post-vaccination in the PPSV23 groups.

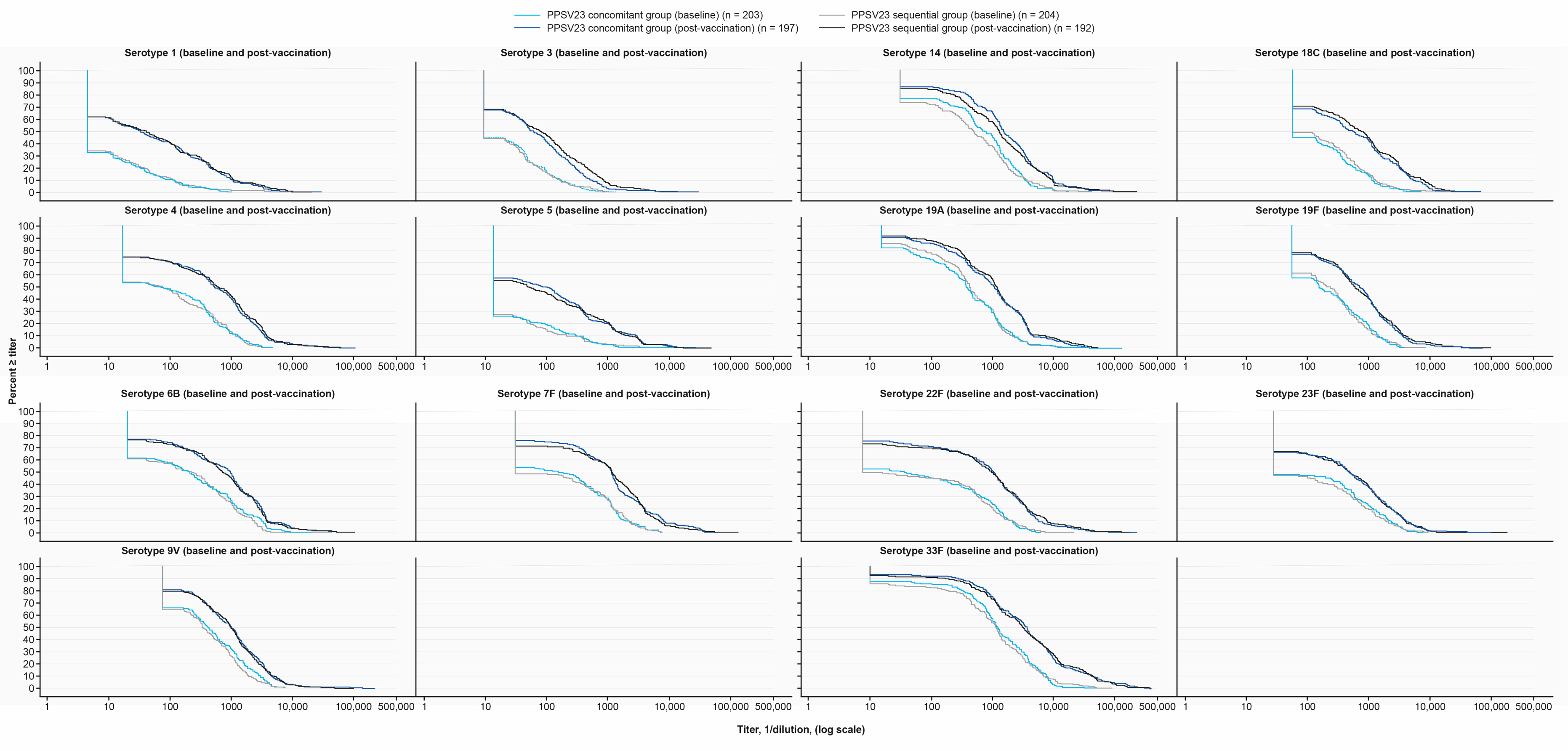

OPA: opsonophagocytic activity; PPSV23: 23-valent pneumococcal polysaccharide vaccine.

Supplementary Figure S2. Reverse cumulative distribution curve of OPA titers at baseline and 30 days post-vaccination in the PCV15 groups.

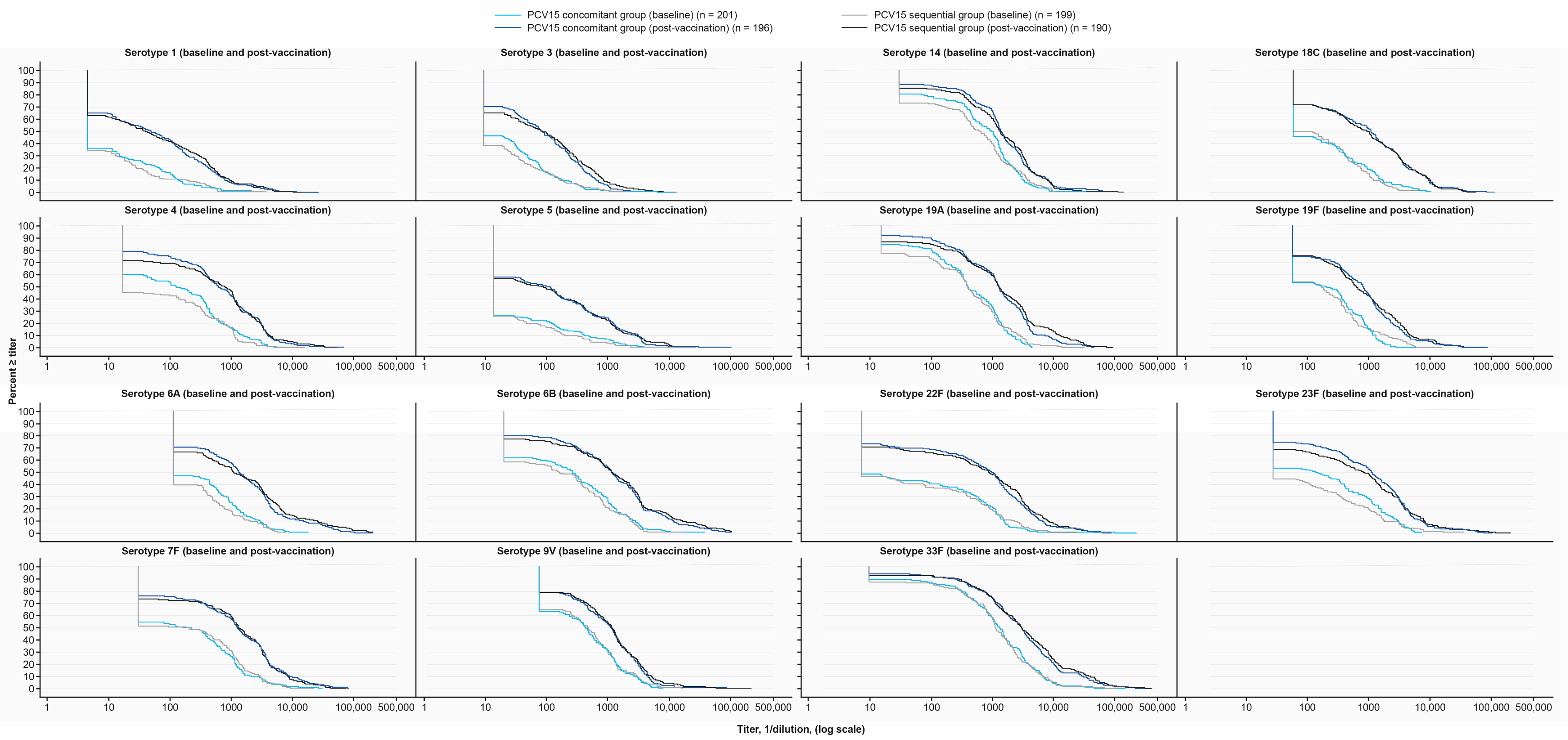

OPA: opsonophagocytic activity; PCV15: 15-valent pneumococcal conjugate vaccine.

Supplementary Figure S3. Reverse cumulative distribution curve of SARS-CoV-2-specific binding titers at baseline and 30 days post-vaccination with mRNA-1273.

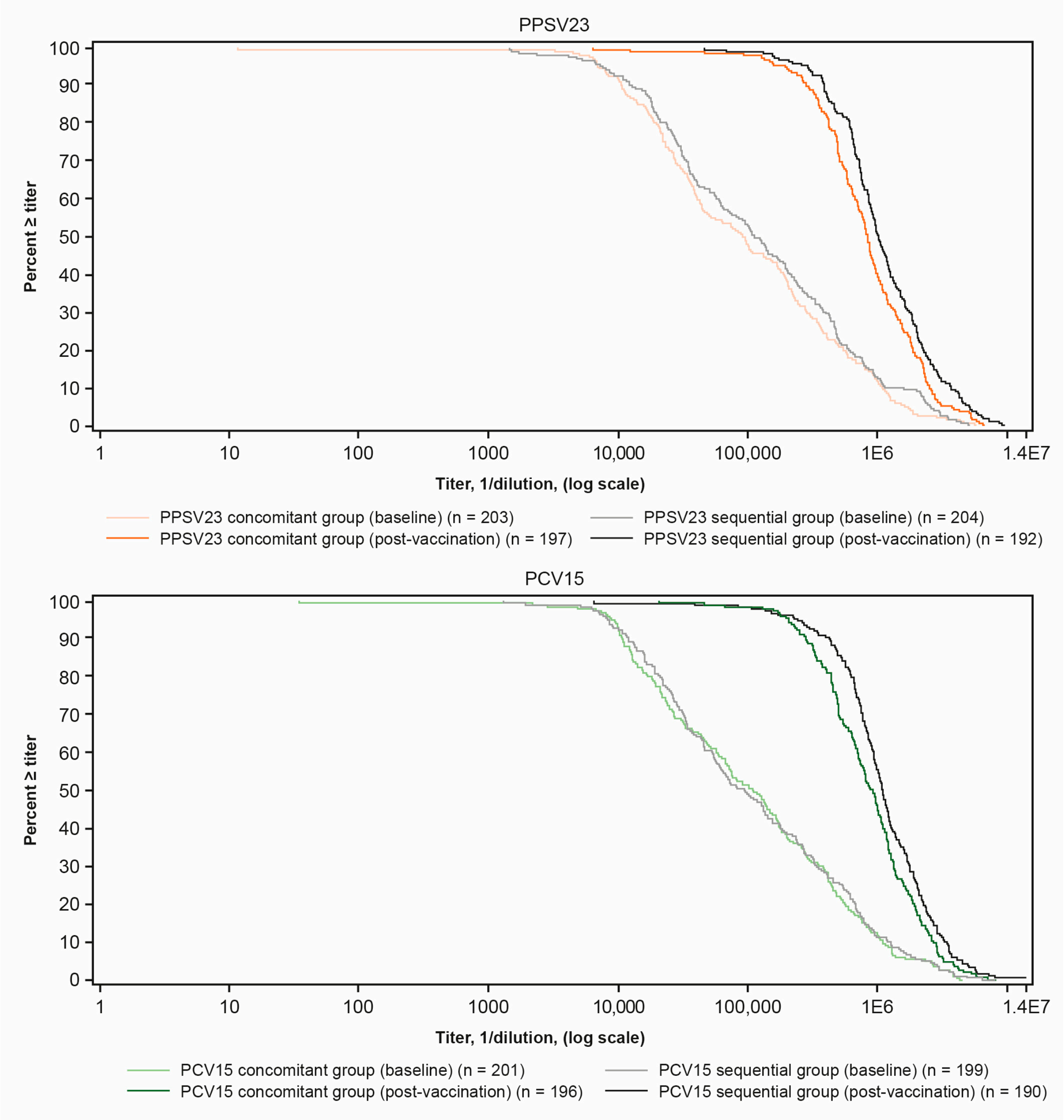

PCV15: 15-valent pneumococcal conjugate vaccine; PPSV23: 23-valent pneumococcal polysaccharide vaccine; SARS-CoV-2: severe acute respiratory syndrome coronavirus 2.
